# Variability in childhood allergy and asthma across ethnicity, language, and residency duration in El Paso, Texas: a cross-sectional study

**DOI:** 10.1186/1476-069X-8-55

**Published:** 2009-12-08

**Authors:** Erik R Svendsen, Melissa Gonzales, Mary Ross, Lucas M Neas

**Affiliations:** 1 University of South Carolina, Arnold School of Public Health, Columbia, SC, USA; 2 University of New Mexico School of Medicine, Department of Internal Medicine, Albuquerque, NM, USA; 3 US Environmental Protection Agency, National Center for Environmental Assessment, Research Triangle Park, NC, USA; 4 US Environmental Protection Agency, Human Studies Division, Chapel Hill, NC, USA

## Abstract

**Background:**

We evaluated the impact of migration to the USA-Mexico border city of El Paso, Texas (USA), parental language preference, and Hispanic ethnicity on childhood asthma to differentiate between its social and environmental determinants.

**Methods:**

Allergy and asthma prevalence was surveyed among 9797 fourth and fifth grade children enrolled in the El Paso Independent School District. Parents completed a respiratory health questionnaire, in either English or Spanish, and a sub-sample of children received spirometry testing at their school. Here we report asthma and allergy outcomes across ethnicity and El Paso residency duration.

**Results:**

Asthma and allergy prevalence increased with longer duration of El Paso residency independent of ethnicity and preferred language. Compared with immigrants who arrived in El Paso after entering first grade (18%), lifelong El Paso residents (68%) had more prevalent allergy (OR, 1.72; 95% CI, 1.32 - 2.24), prevalent asthma (OR, 1.75; 95% CI, 1.24 - 2.46), and current asthma (OR, 2.01; 95% CI, 1.37 - 2.95). Spirometric measurements (FEV_1_/FVC and FEF_25-75_) also declined with increasing duration of El Paso residency (0.16% and 0.35% annual reduction, respectively).

**Conclusion:**

These findings suggest that a community-wide environmental exposure in El Paso, delayed pulmonary development, or increased health of immigrants may be associated with allergy and asthma development in children raised there.

## Background

Asthma is the most commonly diagnosed chronic disease in childhood [1-3]. The prevalence of allergy and asthma related outcomes have been shown to vary across race and ethnicity, with Hispanics having the lowest prevalence of asthma in the United States (U.S.) [4-7]. However, Hispanic ethnicity includes numerous subgroups that differ by country of origin, language preference, duration of residence in the U.S., and asthma prevalence [8,9]. Of particular interest are the populations living in the four U.S. states bordering Mexico[10,11], which comprise 56% of the total Hispanic and 72% of the Mexican American population in the United States[12] which has the lowest prevalence of asthma of all U.S. Hispanic subgroups [13]. This population includes monolingual English and Spanish speakers, bilingual individuals, and both immigrant, and non-immigrant Hispanics. There are some population-based epidemiologic studies of the heterogeneity of asthma within subgroups of the Mexican-American population[4,7,14-21], but very few specifically across language preference and duration of residence [22-26].

Migrant studies compare differences in health outcomes between a population originating from a study area with another population that has migrated into that same study area[27,28]. Such studies have been effective hypothesis-generating tools for separating the relative genetic and environmental contributions to disease, including asthma[28]. Migration has also been used to investigate the role of changes in air pollution exposure on pulmonary function growth levels in children[29]. Because Mexican-American populations differ across acculturation and language preference, migrant studies could help identify the differential social and environmental determinants of disease within these populations.

El Paso, Texas is a city of nearly 700 thousand people, 78% of whom are Hispanic[12] of predominantly Mexican ancestry. El Paso is located across the international border from the Mexican city Ciudad Juárez, population 1.2 million[30]. El Paso also has a large military installation with a relatively transient population. El Paso and Ciudad Juárez are located in the Paso del Norte Airshed, which is geographically isolated and has a history of poor air quality [31-42] primarily due to the local generation of air pollutants from mobile sources. Therefore, El Paso serves as a good population to examine the health effects of locally generated air pollution within a migrant study.

We studied the environmental determinants of childhood asthma among children ages 9-11 in El Paso, Texas using a school-based cross-sectional survey. To investigate the long-term health effects of living in El Paso, here we compared children across El Paso residency duration and ethnicity to separate the social from the environmental determinants of allergy and asthma during child development.

## Methods

### Parent Study

In February, 2001, 9797 fourth and fifth grade students enrolled in the 54 elementary schools of the El Paso Independent School District received a survey packet for their parents or guardians to complete. The packet contained both an English- and a Spanish-language respiratory health questionnaire and consent form for spirometry testing, which was completed in the parent or guardian's preferred language The ten-page questionnaire conformed to a standard respiratory questionnaire[43]. Spirometry testing was performed between March and May 2001 at a subset of 20 elementary schools which were selected based on their geographic distribution and without prior knowledge of children's health conditions. Selected schools were randomized to one of six study weeks and one of three field teams. Spirometry tests used American Thoracic Society certified spirometers (SensorMedics 922; Cardinal Health, Dublin, OH), and were performed during normal school hours using standardized protocols for children which account for the complexity of testing children, including quality control and assurance procedures [44,45]. Because of our relatively large sample size, we used 3 clinical teams with two separate sets of equipment to test all the children. Prior to each test, we verified receipt of a signed parental consent form, obtained the informed assent of the child, and measured their weight and standing height. We excused from spirometry testing those children who had smoked more than five cigarettes in their lifetime or who had a respiratory infection within the previous two weeks. The study protocol was reviewed and approved by the University of North Carolina School of Medicine's Institutional Review Board and by the Human Subjects Research Review Official for the US Environmental Protection Agency.

### Outcome Assessment

The health outcomes evaluated include prevalent allergy, prevalent asthma, current asthma, and several spirometry measures. A child was considered to have prevalent allergy if he or she had a parental report of a physician's diagnosis of allergy or if the parent reported any specifically diagnosed allergy to foods, house dust or house dust mites, pollens, molds, animal fur or dander, insect bites or stings, feathers, skin contact irritants (other than poison ivy, oak or sumac), or other allergens. Prevalent asthma was defined as a parental report of a physician's diagnosis of asthma. If a child had ever been diagnosed by a doctor with asthma then the parent was directed to answer further questions about their asthma such as medication use. Because some parents may have believed that their child may have outgrown their asthma, we defined current asthma as prevalent asthmatics who additionally either had any asthma exacerbations or taken any asthma medications during the previous year. Spirometry measures used include forced vital capacity (FVC), forced expiratory volume in the first second (FEV_1_), peak expiratory flow (PEF), forced expiratory flow in the middle half of the expiration (FEF_25-75_), and the ratio of forced expiratory flow in the first second to forced vital capacity (FEV_1_/FVC). We used deficits in FEV_1_/FVC and FEF_25-75 _as indicators of asthma development. We calculated the best spirometry values for analysis by averaging the measures from the two maneuvers with the first and second greatest FVC. Symptoms of asthma (ever had wheeze, wheeze without cold, wheeze with cold, chronic wheeze) and allergy (ever had hay fever, hay fever in the past year, doctor visit for hay fever in past year) were also assessed.

### Duration of El Paso Residency

Our primary exposure metric was El Paso residency, as queried by the question "How long has this child lived in this city? Has lived in this city since birth, Moved to this city before the age of 2, Moved to this city when 2 years or older but before starting first grade, Moved to this city in first grade or later but not in the last 12 months, Moved to this city in the last 12 months, Don't know". El Paso residency was then stratified into lifelong resident, early immigrant children (defined as children who arrived before entering first grade), and late immigrant children (defined as children who arrived after entering first grade), which served as the referent group. Similar strata were used separately to assess duration of residence in the child's current neighborhood (within 1 mile (2 km) of your current residence) and current residence. No additional information about the child's or family's international or domestic migration to El Paso was collected.

### Exposure Assessment

Air pollution in El Paso improved during the lifetime of study children. For this paper, modeled peak season mobile source air pollution was included as a covariate to adjust for the effects of air pollution exposure within the last year. The methods that were used to estimate local exposure to mobile source air pollution during the peak months have been previously reported elsewhere[32-35,38,46-49]. In brief, indicators of mobile source emissions were measured at the same elementary schools where children received spirometry and then spatially interpolated over the study area to get personal estimates of mobile-source air pollution exposure during the peak winter season. We investigated the additional queried potential indoor home environmental risk factors of pesticide use in the past year, reported cockroach problem, reported mouse problem, pet dog, pet cat, air conditioning, and a gas stove with a continuously burning pilot light for only their current residence. No such data were collected for prior residences.

### Ethnicity

Hispanic ethnicity was queried with standard questions[50] and stratified into three groups: non-Hispanic children, Hispanic children whose parent/guardian completed the English-language questionnaire (Hispanic/English), and Hispanic children whose parent/guardian completed the Spanish-language questionnaire (Hispanic/Spanish).

### Data Analysis

Descriptive statistics were generated for our primary outcomes and exposures after stratification across ethnicity and El Paso residency groups. Chi-square tests were performed to assess trends and differences between strata. Final data analyses were performed with SAS version 9.2 software (SAS Institute Inc., Cary, North Carolina) using generalized estimating equations with mixed effect logistic (PROC GENMOD) and linear (PROC MIXED) regression procedures with model-based standard error estimates. Mixed models with a random effect of clinical team * equipment set were used in the final models to adjust for any unmeasured local neighborhood, technology, clinical team, or location of spirometry exam effects. Covariates which were associated with the outcomes at a p < 0.1 level were considered for inclusion in final models. All confounders were considered as effect modifiers, also, but only the first-order interaction of extended stay outside El Paso during the past year (> 6 weeks) with lifelong El Paso resident and a first-order interaction of Hispanic ethnicity with immigration into El Paso were included in final models (p < 0.05). The final most parsimonious multivariate logistic regression models also included the fixed effects of sex, ethnicity group, maximum parental education level, single parent, household smoker, parental history of allergy and/or asthma. Spirometry measurements were log transformed and adjusted for the random effects of field team and spirometer in addition to the fixed effects used in the logistic models (without the interactions), age, child height (log transformed), child weight (log transformed), and an interaction of sex with height. We did not adjust for body mass index because inclusion of that variable did not improve the model fit and height and weight were already included in the model. Hispanic ethnicity significantly modified the association of both weight and height with pulmonary function measures (p < 0.05), so we adjusted all of our final spirometry measure models accordingly. Exclusions from data analysis included children who had extreme heights (< 120 cm or > 160 cm) or weights (< 22.7 kg or > 68.1 kg), children with severe illnesses or medical conditions (cystic fibrosis, chest operation, severe chest injury, heart conditions, or on oxygen after birth for two or more weeks) and children of extreme age for their grade level (under nine years or over 12 years).

## Results

### Selection

**Figure 1:**
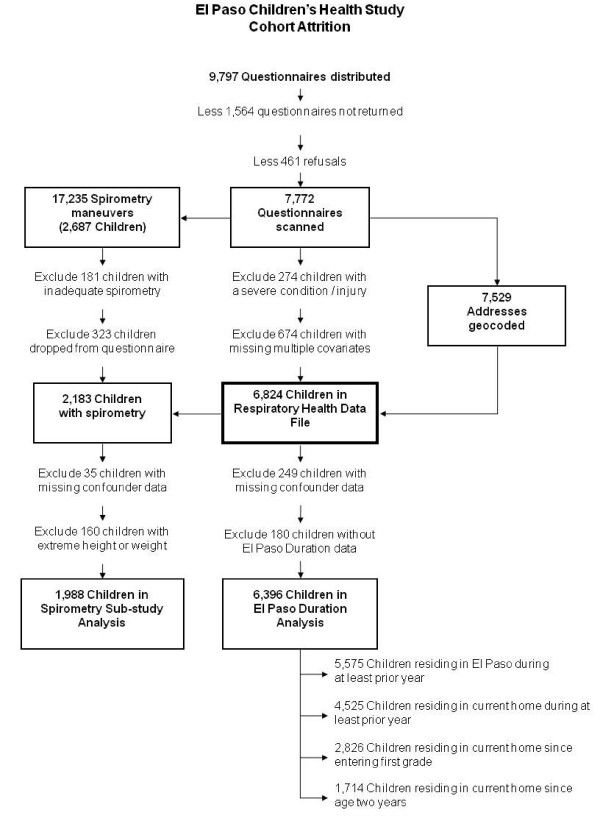
**Cohort attrition within the El Paso Children's Health Study**. El Paso Independent School District, February 2001.

Of the 9797 students who received questionnaire packages, 79% completed and returned them (Figure 1). Analyses were limited to the analytical group of 6396 children with complete data. Of the 2687 pulmonary function exams performed, 1988 children (74%) met all the inclusion criteria with an average of 3.7 (range, 3 - 8) maneuvers per child, which represented 31% of the above analytical group.

The majority of children were Hispanic (81%) and had lived in El Paso their entire lifetime (68%). About one third of study children were diagnosed with allergies (32%), 12% with asthma. Ten percent currently had asthma. Children who underwent spirometry testing were similar to children not selected based on their El Paso residency duration, prevalent allergy, both current and prevalent asthma, all demographics, and anthropometrics (p > 0.1). Characteristics of our study population stratified by ethnicity/language preference and duration of El Paso residency are presented in Tables 1 and 2.

**Table 1:** Selected factors of children enrolled in the El Paso Children's Health Study by Hispanic ethnicity.

	Non-Hispanic	Hispanic	
		**English Preferred**	**Spanish Preferred**

	**(n = 1226)**	**(n = 3005)**	**(n = 2165)**

	**%**	**%**	**%**

El Paso Resident Since:			

Birth	42.9	76.9	70.8

Before entering 1st grade	19.5	11.3	13.2

After entering 1st grade	37.6	11.8	16.0

Maximum Parental Education:			

Some secondary school	2.0	10.1	33.6

High School Graduate	11.5	22.6	22.2

Some Post-High School	18.6	19.4	30.5

College Graduate or Higher	67.9	47.9	13.7

Outcomes:			

Prevalent Allergy	38.9	36.9	22.0

Prevalent Asthma	15.7	13.9	7.8

Current Asthma	12.2	11.8	5.9

Covariates:			

Female	49.5	50.9	52.2

Parental Allergy	53.3	40.9	13.8

Parental Asthma	19.3	13.7	7.9

Smoker in household	29.1	29.8	36.2

Unadjusted prevalence of Non-Hispanic, Hispanic English-preferred, and Hispanic Spanish-preferred ethnicity in 4th or 5th grade children: El Paso Independent School District, February 2001 (n = 6396)

**Table 2:** Selected factors of El Paso Children's Health Study participants by El Paso nativity.

	Lifelong	Immigrant	
		**Early: Before entering 1^st ^grade**	**Late: After entering 1^st ^grade**

	**(n = 4370)**	**(n = 862)**	**(n = 1164)**

	**%**	**%**	**%**

Non-Hispanic	12.0	27.7	39.6

Hispanic, English Preferred	52.9	39.2	30.5

Hispanic, Spanish Preferred	35.1	33.1	29.9

Maximum Parental Education:			

Some secondary school	18.7	13.7	10.3

High School Graduate	22.7	17.9	13.4

Some Post-High School	22.4	22.7	25.4

College Graduate or Higher	36.2	45.7	50.9

Outcomes:			

Prevalent Allergy	33.0	33.5	28.5

Prevalent Asthma	12.6	12.2	10.5

Current Asthma	10.5	9.6	7.8

Covariates:			

Female	51.4	50.2	50.2

Parental Allergy	32.3	39.2	37.1

Parental Asthma	12.4	13.9	13.4

Smoker in household	32.6	31.9	29.0

El Paso nativity- duration of El Paso residence by 3 strata. El Paso Children's Health Study of 4th or 5th grade children: El Paso Independent School District, February 2001 (n = 6396)

### Study Population Characteristics stratified by ethnicity/language and residency duration

Hispanic children, regardless of their parent's language preference, were predominantly lifelong El Paso residents (> 70%). The English-speaking parents, regardless of Hispanic ethnicity, reported higher education attainment than the parents of the Hispanic/Spanish children (Table 1). Parental education, also, varied by duration of El Paso residency (Table 2). Children from the Hispanic/Spanish group had the lowest prevalence of allergies, asthma (current and lifetime) and parental allergy and parental asthma while children from the Non-Hispanic group had the highest (p-trend < 0.001). The unadjusted prevalence of pulmonary health outcomes varied across the three ethnicity/language groups. These groups did not differ by child's age or the total number of years the child had lived in El Paso (p > 0.1). However, the unadjusted prevalence of allergy and asthma were higher among lifelong El Paso residents than among immigrants, with duration-dependent effects observed in both current and prevalent asthma (Table 2). Similar patterns were not found with duration of residence in the same neighborhood or the same home (p > 0.1).

### Prevalence of Asthma and Allergy

**Figure 2:**
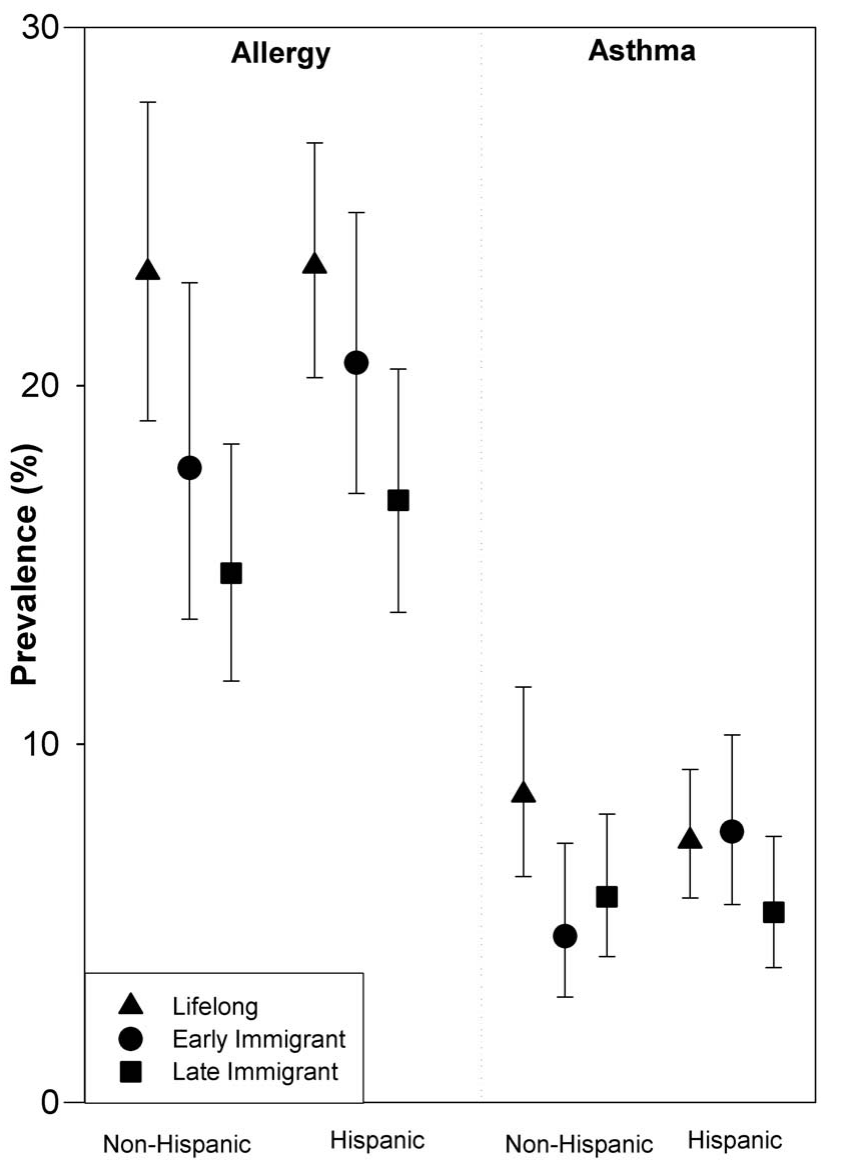
**The association of El Paso nativity with the prevalence of allergy and asthma**. El Paso Children's Health Study of 4^th ^or 5^th ^grade children: El Paso Independent School District, February 2001 (n = 6396). Models were adjusted for potential confounders using mixed effects models with no intercepts. The fixed effects include questionnaire language, socio-economic status (maximum parental education level, single parent), duration of El Paso residency (nativity), environmental tobacco smoke (parental smoker), parental history (allergy, asthma), the first-order interaction of extended stay outside El Paso within past year (> 6 weeks) with duration of El Paso residency, and the first-order interaction of Hispanic ethnicity with duration of El Paso residency.

After adjustment, the pattern of increasing prevalence of allergy with increasing duration of El Paso residency followed a similar pattern in both Hispanic and non-Hispanic groups (Figure 2). Among lifelong El Paso residents, non-Hispanic children had more asthma, but not allergy, than Hispanic children (Figure 2). The associations of El Paso residency with increased asthma and allergy outcomes were not confounded or modified by modeled peak season ambient mobile source air pollution indicators, neighborhood or home-specific exposures, or indoor environmental risk factors (p > 0.1). The main effects of modeled peak season ambient mobile source air pollution indicators on asthma and lung function outcomes will be presented separately.

After adjustment, asthma and allergy prevalence were slightly lower in both non-Hispanic and Hispanic/Spanish children compared to Hispanic/English children (Table 3). The established risk factors of male sex, parental allergy, and parental asthma history were independently associated with both asthma and allergy prevalence in the adjusted models (p < 0.05). Lifelong El Paso resident children were more likely to have been diagnosed with allergies (72%), asthma (75%), and current asthma (101%) than those who had moved into El Paso since entering first grade (Table 3, adjusted models). We found similar associations with symptoms of asthma and allergy (p < 0.1). The Hispanic ethnicity patterns of asthma and allergy seen with the children (Table 3) were present in their parents' asthma and allergy, also (p < 0.05). However, the prevalence of allergy and asthma in parents did not increase with increasing duration of residence in El Paso during their child's lifetime (p > 0.1). Extended stay outside El Paso within the past year (> 6 weeks) significantly modified the El Paso residency duration association with diagnosed allergy (p < 0.05), but not on diagnosed or current asthma.

**Table 3:** Adjusted associations of Hispanic ethnicity/questionnaire language and El Paso nativity with asthma and allergy.

	Diagnosed Allergy			Diagnosed Asthma			Current Asthma		
Crude Model*	**OR**	**95%CI**		**OR**	**95%CI**		**OR**	**95%CI**	

Hispanic ethnicity/questionnaire language									

Hispanic, Spanish Language	0.53	0.47	0.60	0.53	0.44	0.64	0.48	0.39	0.59

Hispanic, English Language	1.00			1.00			1.00		

Non-Hispanic, English Language	1.14	0.99	1.32	1.25	1.03	1.52	1.15	0.93	1.43

El Paso Nativity: Duration of Child's El Paso residence									

Lifelong	1.34	1.15	1.57	1.37	1.10	1.70	1.49	1.17	1.91

Early Immigrant: Since before entering 1^st ^grade	1.31	1.08	1.59	1.25	0.94	1.65	1.31	0.96	1.79

Late Immigrant: Since after entering 1^st ^grade	1.00			1.00			1.00		

Adjusted Model**									

Hispanic ethnicity/questionnaire language									

Hispanic, Spanish Language	0.80	0.69	0.93	0.78	0.63	0.96	0.70	0.55	0.88

Hispanic, English Language	1.00			1.00			1.00		

Non-Hispanic, English Language	0.84	0.68	1.05	0.85	0.63	1.15	0.73	0.52	1.03

El Paso Nativity: Duration of Child's El Paso residence									

Lifelong	1.72	1.32	2.24	1.75	1.24	2.46	2.01	1.37	2.95

Early Immigrant: Since before entering 1^st ^grade	1.26	1.03	1.55	1.16	0.87	1.55	1.22	0.88	1.69

Late Immigrant: Since after entering 1^st ^grade	1.00			1.00			1.00		

Coded as the prevalence of ever diagnosed allergy, ever diagnosed asthma, and current asthma by Hispanic ethnicity/questionnaire language and duration of El Paso residence (nativity), El Paso Children's Health Study of 4th or 5th grade children: El Paso Independent School District, February 2001 (n = 6396)

* Mixed effects model with random school-specific intercepts

** Mixed effects model with random school-specific intercepts and adjusted for the fixed effects of socio-economic status (maximum parental education level, single parent), environmental tobacco smoke (parental smoker), parental history (allergy, asthma), the first-order interaction of extended stay outside El Paso within past year (> 6 weeks) with duration of El Paso residence, and the first-order interaction of Hispanic ethnicity with duration of El Paso residence

We further examined the effect of El Paso residency duration on the patterns of parent-reported diagnosed allergies to investigate whether local allergens might partially explain the associations reported above. Compared to the other groups, Hispanic/Spanish children had fewer allergies to all aeroallergens except feathers (Table 4). Forty six percent of the children were allergic to only one type of allergen. Pollen was the most common allergen reported. Few children were only allergic to pets (0.7%). Allergy to pollen only increased monotonically with increasing duration of El Paso residency while allergy to animal fur or dander only did not (Table 4). After including the same predictors in the adjusted models from Table 3 and random school effects, lifelong El Paso residence was significantly associated with allergy to pollens (Odds Ratio (OR), 1.44; 95% Confidence Interval (CI), 1.09 - 1.91). These associations were robust to further adjustment for modeled peak season mobile-source air pollution, or potential indoor risk factors (p < 0.05).

**Table 4:** El Paso nativity and ethnicity/questionnaire language groups by queried diagnosed allergy type.

	El Paso Nativity			Ethnicity		
	Lifelong	Immigrant		Non-Hispanic	Hispanic	
		Early: Before entering 1^st ^grade	Late: After entering 1^st ^grade		English Preferred	Spanish Preferred
	(n = 4370)	(n = 862)	(n = 1164)	(n = 1226)	(n = 3005)	(n = 2165)
	%	%	%	%	%	%
Diagnosed allergy to:						

things ingested (e.g. food or medicine)	5.2	5.5	4.8	7.5	5.3	3.7

house dust or dust mites	11.7	12.8	10.2	12.6	14.4	7.1

pollens (e.g. grasses, trees, flowers)	24.7	22.2	19.8	28.1	28.1	14.5

molds	4.9	5.7	5.6	7.8	6.4	2.0

animal fur or dander (e.g. cats or dogs)	8.9	8.5	6.4	10.1	9.3	6.3

insect bites or stings (e.g. bee stings)	2.9	2.1	2.2	3.5	3.0	1.9

feathers (e.g. pillows or comforters)	1.8	2.2	1.7	1.6	1.7	2.0

things that contact the skin* (e.g. wool)	2.0	3.3	1.9	3.2	2.2	1.6

other	2.7	2.2	2.2	2.9	2.8	1.9

unknown	2.7	3.25	2.5	3.7	3.5	1.1

only pollens	8.6	7.8	6.3	9.1	9.6	5.4

only animal fur or dander	0.7	0.5	0.6	1.0	0.5	0.8

only non-pollen aeroallergens**	12.9	14.0	11.9	15.3	15.7	7.7

Coded as ever diagnosed allergy, Hispanic ethnicity/questionnaire language and duration of El Paso residence (nativity), El Paso Children's Health Study of 4th or 5th grade children: El Paso Independent School District, February 2001 (n = 6396)

*not poison oak, ivy, or sumac

**aeroallergens: house dust or house dust mites, pollens, molds, animal fur or dander, or feathers

**Figure 3:**
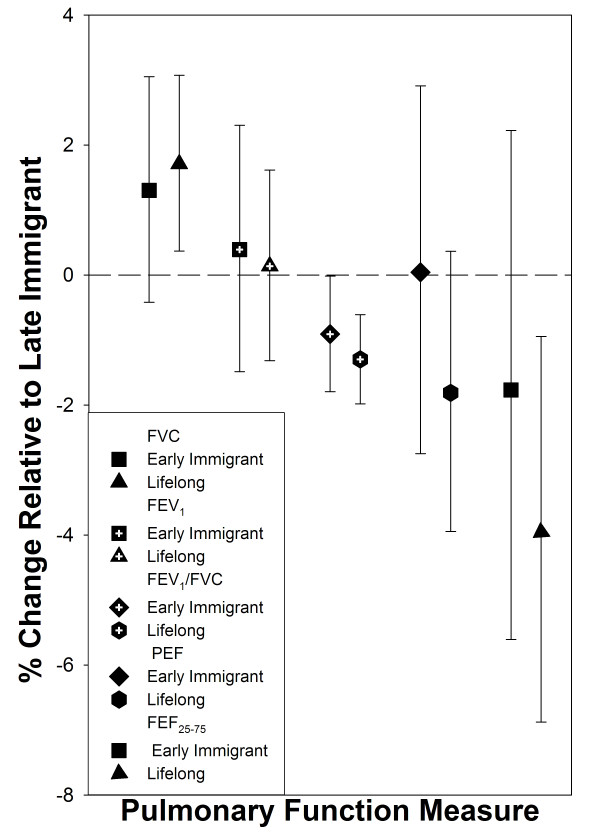
**The association of El Paso nativity with pulmonary function**. El Paso Children's Health Study data are stratified by Hispanic ethnicity, in 4^th ^or 5^th ^grade children: El Paso Independent School District, Spring 2001 (n = 1988). El Paso nativity- 3 duration of El Paso residence strata, % change in pulmonary function measures are relative to late El Paso Immigrants- with random team/instrument intercepts and adjusted for the fixed effects of age, sex, race, ethnicity, language preference, log of child's height, log of child's weight, interaction of sex with child's height, interaction of Hispanic ethnicity with child's height, interaction of Hispanic ethnicity with child's weight, parental history (allergy, asthma), environmental tobacco smoke (household smoker), and socio-economic status (maximum parental education, single parent).

We then analyzed spirometric measures across El Paso residency duration categories. Residency duration was independently associated with measures of small airway obstruction in a dose-dependent fashion (Figure 3). We found that FEV_1_/FVC decreased monotonically with increasing residency duration, with a 0.9% decrease (95% CI, -1.8 - -0.0) among children who had lived in El Paso since before entering first grade and a 1.3% decrease (95% CI, -2.0 - -0.6) among children who were lifelong El Paso residents. Lifelong residents had a 4.0% decrease (95% CI, -6.9 - -0.4) in FEF_25-75 _compared with late El Paso immigrant children, while no such associations were found for FEV_1 _and PEF. Inclusion of potential home environmental risk factors or modeled peak season mobile-source air pollution did not modify this pattern of spirometry measures results.

**Figure 4:**
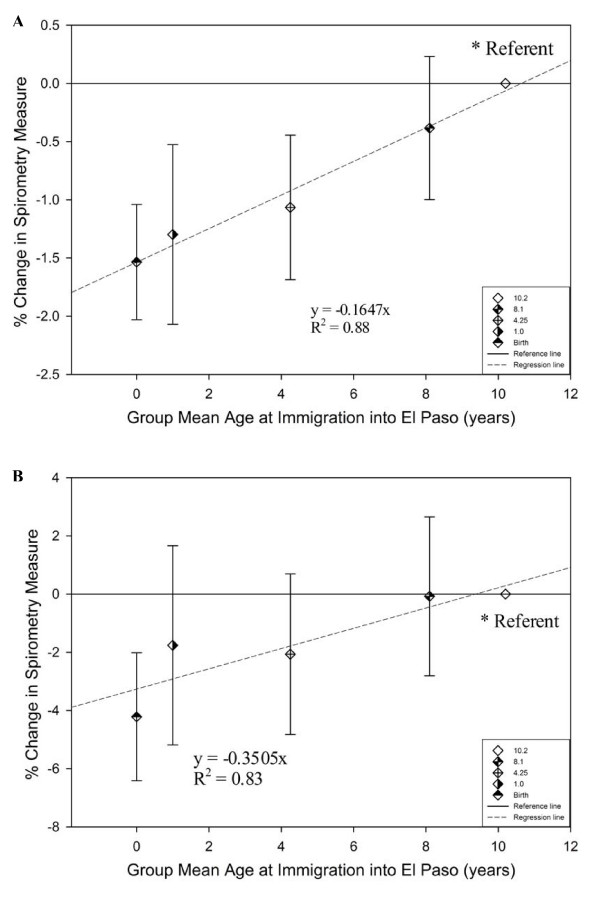
**A and B - Linear decrement in FEV_1_/FVC and FEF_25-75 _by stratum-specific mean age at immigration into El Paso**. In 4^th ^or 5^th ^grade children:% change in spirometry outcome measure relative to immigrants into El Paso in the last year- with random team/instrument intercepts and adjusted for the fixed effects of age, sex, race, ethnicity, language preference, log of child's height, log of child's weight, interaction of sex with child's height, interaction of Hispanic ethnicity with child's height, interaction of Hispanic ethnicity with child's weight, parental history (allergy, asthma), environmental tobacco smoke (household smoker), and socio-economic status (maximum parental education, single parent). El Paso Independent School District, Spring 2001 (n = 1988).

Lastly, we explored the linearity of the monotonic trend in decreasing FEV_1_/FVC and FEF_25-75 _with increasing El Paso residency duration. Children were stratified into new El Paso residency strata based on whether they had moved to El Paso a) within the past year, b) since entering first grade but before the past year, c) since age two years but before entering first grade, d) before age two years but after birth and e) since birth. We then calculated the mean age at immigration into El Paso for each stratum based on the child's age at entry into first grade (10.2, 8.1, 4.25, 1.0, and 0, respectively). Children who moved into El Paso during the past year served as our reference group. We then modeled El Paso residency with these five strata using models otherwise identical to those used previously. FEV_1_/FVC decreased linearly, with a 0.16% reduction with each additional year of residence in El Paso (R^2^= 0.88), as did FEF_25-75 _with a 0.35% annual reduction (R^2^= 0.83) (Figure 4).

## Discussion

In our study population of Non-Hispanic and Hispanic children of predominantly Mexican-American heritage, we found that asthma and allergy prevalence were slightly lower in both non-Hispanic and Hispanic/Spanish-language preferred children compared to Hispanic/English-language preferred children. Also, diagnosed allergy, diagnosed asthma, and current asthma prevalence were significantly higher in lifelong El Paso resident children than those who had moved into El Paso since entering first grade. Similarly, El Paso residency duration was independently associated with measures of small airway obstruction in a dose-dependent fashion. All of these associations were robust to further adjustment for modeled peak season mobile-source air pollution, or potential indoor risk factors (p < 0.05).

Heterogeneity in asthma and allergy between Non-Hispanic and Hispanic persons has been well documented[3,4,20,51]. Our measure of asthma prevalence in Hispanic/Spanish children was similar to that found in a sister study in neighboring Ciudad Juárez, Mexico[52,53]. Klinnert, et al., reported demographic and environmental differences between acculturated and non-acculturated (as measured by language preference) Hispanics, predominantly of Mexican ancestry, in a clinical cohort at risk for asthma in the Southwestern United States[11]. Further research is needed to better characterize how Hispanic sub-cultures within the southwestern United States differ in their exposures and allergic and asthmatic health outcomes.

Deficits in FEV_1_/FVC or FEF_25-75 _in the absence of FEV_1 _and PEF deficits (e.g. below the lowest limit of normal[54]) but with normal lung volume (FVC) can be used as indicators of small airway obstruction, while PEF and FEV_1 _deficits with normal lung volume (FVC) alone can be used as indicators of larger airway obstruction[55]. Small airway obstruction in the absence of acute asthma exacerbations can be an indicator of recently developed asthma or other early obstructive airways disease while larger airway obstruction in the absence of an acute exacerbation can be an indicator of more advanced or longer-term asthma or obstructive airway disease[55]. Therefore, our results suggest that increasing duration of El Paso residency is associated with small airway obstruction, or possibly subtle fibrosis in the lung interstitium (scarring from repeated injury/inflammation) which can also be present with decreased FEV_1_/FVC and FEF_25-75_. The Southern California Children's Health Study has shown that exposure to air pollution in childhood reduces lung development[29]. An alternative explanation could be that the duration of El Paso residency effects on lung function may be due to reduced functional development over time. However, yet another explanation could be that the El Paso children are simply normal and that the rural children who migrate into El Paso are healthier than normal [56-62]. Subramanian, *et al*.[20], found that foreign born children were 74% less likely to have been diagnosed with asthma than those born in the United States. Eldeirawi, et. al [63-65], found similar results, and with immigrant acculturation[64]. We do not have any data on how rural previous residences were. However, given the proximity of El Paso to Ciudad Juárez, Mexico, it is reasonable to assume that many of the immigrants into El Paso may have come from or through that city. Further study is needed to determine if the duration of El Paso residence effects on asthmatic health outcomes is a real disease progression effect resulting from a localized environmental exposure, a delayed pulmonary development effect, or simply artifact of children migrating from rural areas.

However, interestingly, March (peak season for Mulberry pollen) was the most frequently reported month for peak asthma symptoms in the group of children who had only allergy to pollens (data not shown). Mulberry allergy is very common in the desert southwest [66-68] and Mulberry pollen can profoundly reduce air quality during peak pollen season[67]. We only used air pollution exposure data from November, entirely missing the primary pollen season in El Paso (spring). Particulate matter air pollution has been reported to act as an adjuvant, binding and transporting allergenic proteins [69-71]. Such exposures are reportedly associated with adverse pulmonary and allergic health outcomes[72]. Perhaps the interaction of diesel exhaust particles with local allergens may explain our observed El Paso residency effects on allergy. Further study is needed to determine if locally common pollen (e.g. Mulberry) modifies the effect of locally-generated diesel exhaust particulates on the development of pulmonary and allergic health problems in children.

**Figure 5:**
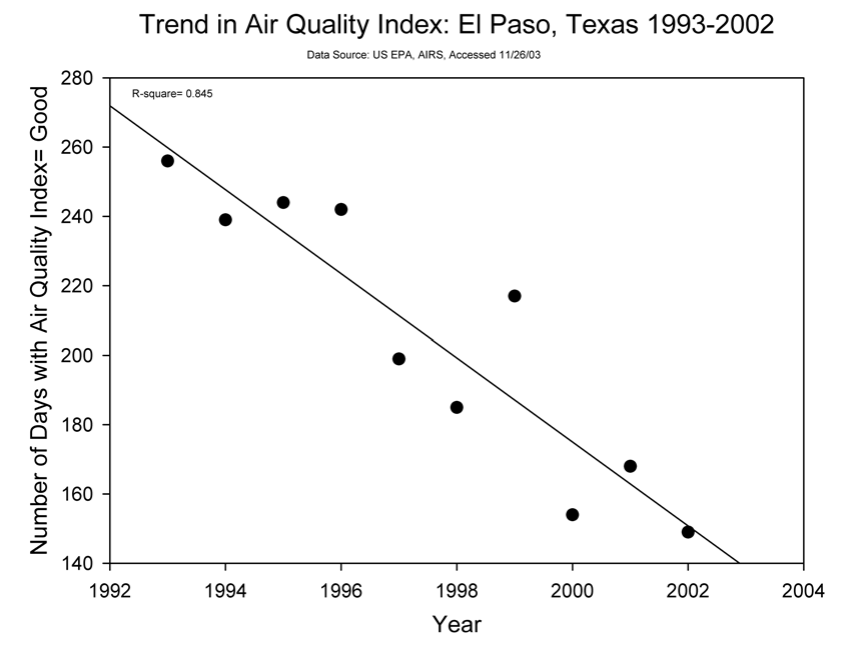
**Trend in EPA's Air Quality Index = Good for 1993-2002, El Paso, Texas**.

Air pollution in El Paso is, and has been for many years, a significant public health concern[32,73-79]. However, our indicator of peak season mobile-source air pollution did not modify the El Paso residency association with asthma and allergy outcomes (p > 0.1). From the early 1990s through early 2000s there was a linear decrease in the number of days that the EPA's Air Quality Index for El Paso was Good (Figure 5) while there was a concurrent decrease in the annual mean concentration of ozone[80]. The air pollution that we modeled may not be the only air pollution measures associated with adverse health outcomes in El Paso due to prolonged residency. Perhaps it is not just the peak concentration or even the annual average, but the exposure to elevated air pollution more frequently throughout the year or the peak pollen period which drives the duration of El Paso residency effect. Further study is needed to determine what explains the duration of El Paso residency effects on asthmatic health outcomes.

However, our study had some additional limitations. This study was cross-sectional, and has limitations by design in understanding the causality of our exposure of interest. We were only able to partially adjust for socio-economic status by using the surrogate of parental education level, which may mean that some of the language/ethnicity effect could be explained by yet unmeasured indicators of socio-economic status. We did not ask questions about the child's physical fitness or access to medical care. Both of physical fitness and access to medical care may be associated with both immigration status and asthma and allergy prevalence. Therefore, we cannot rule out poor physical fitness in lifelong El Paso residents nor poor access to diagnostic care with migrants as possible explanations for our asthma and allergy prevalence findings. This study was designed to assess the effects of outdoor air pollution on health, not indoor or near-roadway effects. Therefore, our measures of potential indoor air exposures were relatively imprecise, potentially biasing those results towards the null, and we were unable to assess the additional exposure effects of living within close proximity of a major roadway. However, we believe that our findings are reasonable estimates of the true effects because of our additional findings of El Paso residency effects on spirometry measures. Also, bronchodilator use immediately prior to spirometry testing improves pulmonary function in asthmatics and persons with current airway obstruction. This improvement is most evident in the large airway obstruction measures PEF and FEV_1_. We did not collect information on such medication use, potentially introducing error in our spirometry measurements, especially the large airway obstruction measures. We believe that such short-term medication use would have been unassociated with duration of El Paso residency, allowing for systematic non-differential misclassification that may have underestimated the prevalence of large airway obstruction and reduced our power to detect duration of El Paso residency effects on large airway obstruction. However, despite this, current asthmatics did present with decrements in both flow and volume measures commonly seen in asthmatics (PEF (-2.4%; 95% CI, -5.0 - 0.3), FEV_1 _(-4.7%; 95% CI, -6.7 - -2.7), FEV_1_/FVC (-2.3%; CI, -3.3 - -1.2), FEF_25-75 _(-11%; CI, -14 - -7.6), and FEF_25-75_/FVC (-11%; CI, -15 - -7.5)). Current asthmatics were significantly more likely to have current airway obstruction, as measured by an FEV_1 _> 80% of predicted while FEV_1_/FVC was < 80% (OR, 1.93; CI, 1.29 - 2.90) and FEV_1 _< lowest limit of normal (OR, 2.13; CI, 1.41 - 3.23).

We did not have objective measures of allergic sensitivity, such as allergen skin-prick testing or circulating allergen-specific immunoglobulin E. Many children may not have been tested for allergic sensitization. Some of these untested children may have been allergic. However, it is unlikely that this measurement error would have been differential between lifelong El Paso residents and immigrants. Further studies using clinical measures of allergy detailing the types of local pollen and other allergies are needed to validate the association of increasing allergy in children with increasing El Paso residency duration.

We did not query any information on international immigration or country/city of origin. However, non-Hispanic immigrants, Hispanic/English immigrants, and Hispanic/Spanish immigrants likely moved to El Paso from predominantly different locations. Because asthma and allergy prevalence increased with increasing El Paso residency after adjustment for questionnaire language and ethnicity, having more detailed information on place of origin would not have likely changed the relationship between duration of El Paso residency and allergy or asthma.

## Conclusion

We found that adverse allergic and asthmatic health outcomes increased with increasing duration of residence within El Paso, Texas in fourth and fifth grade children independent of race, sex, or ethnicity, and location within the city in a dose-dependent fashion. El Paso residency may be an indicator of some yet unmeasured ubiquitous environmental exposure/exposures unique to the El Paso/Ciudad Juárez area, delayed pulmonary development, or increased health of immigrants. Further study is needed to elucidate these observations which explain our El Paso residency duration effects and the mechanisms by which such exposures elicit adverse allergic and asthmatic outcomes.

## Abbreviations

(FEV_1_): Forced Expiratory Volume in first second; (FVC): Forced Vital Capacity; (FEF_25-75_): Forced Expiratory Flow in the middle half of the expiration; (PEF): Peak Expiratory Flow; (FEV_1_/FVC): the Ratio of FEV_1 _to FVC; (95%): Ninety five %; (CI): Confidence Interval; (OR): Odds Ratio;

## Competing interests

The authors declare that they have no competing interests.

## Authors' contributions

ES- primary author; designed, analyzed, and reported the findings within this paper. MG- secondary author; co-principal investigator of the parent study, helped design and implement the parent study and helped write portions of this manuscript. MR- tertiary author; helped code and analyze data which supported this paper, and provided editorial comments on the manuscript. LN- principal investigator of the parent study; helped design the sub-study presented in this paper and provided editorial comments on the manuscript. All authors reviewed and approved the last version of the manuscript.
